# Supplementary material for: Open chromatin-guided interpretable machine learning reveals cancer-specific chromatin features in cell-free DNA
Source: Commun Biol. 2025 Nov 12;8:1554. doi: 10.1038/s42003-025-08920-0 (PMC12612109; doi:10.1038/s42003-025-08920-0)
Supplement: Supplementary file 1 — Supplementary Information [file 42003_2025_8920_MOESM1_ESM.pdf]

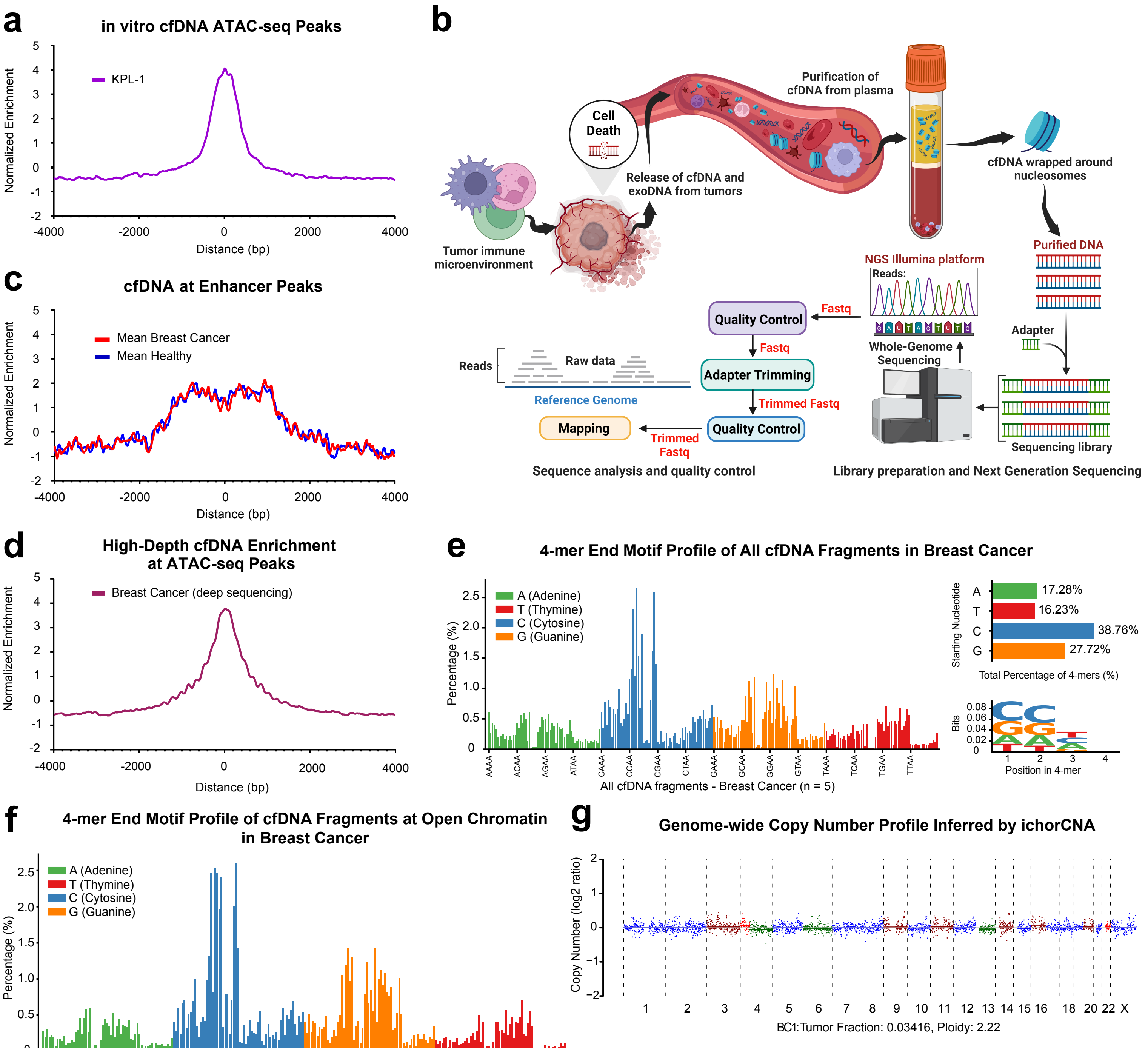

SampleID	NormalCellFraction(n)	TumorFraction	Ploidy
BC1	0.99	0.03416	2.22
BC2	0.99	0.01274	2.2
BC3	0.99	0.00931	2.08
BC4	0.99	0.01091	2.32
BC5	0.99	0.03582	2.21

Supplementary Figure 1. Validation of cfDNA fragment enrichment patterns in vitro and in vivo.

a. Metaplot of in vitro cfDNAs at T47D ATAC-seq peaks. cfDNAs were purified from KPL-1 cell culture medium and analyzed to show enrichment at open chromatin. **b.** Workflow Overview. cfDNAs were isolated from plasma, followed by library preparation and next-generation sequencing. **c.** Comparison of cfDNA enrichment at breast cancer enhancers between breast cancer patients and healthy individuals. **d.** Metaplot displaying in vivo breast cancer cfDNA enrichment at T47D ATAC-seq peaks generated from the deep sequencing data. Approximately 117 million uniquely mapped, PCR duplicate-filtered reads were collected, and their enrichment was assessed at T47D ATAC-seq peaks. **e.** Fragment end motif analysis in breast cancer cfDNAs. Bar plot displays the frequency distribution of all possible 4-mer sequences at the 5′ ends of cfDNA fragments. Motif enrichment logos and the percentage of 4-mers beginning with each nucleotide are shown in the right panels. **f.** 4-mer end motif profiling of cfDNA fragments specifically located at open chromatin (ATAC-seq peaks). The base composition and motif logos are shown for cfDNA derived from open chromatin loci. **g.** Genome-wide copy number profile of a cfDNA sample inferred using the ichorCNA tool. The upper panel shows log2 copy number ratios. The lower table summarizes estimated tumor fractions and ploidy values for five breast cancer cfDNA samples.

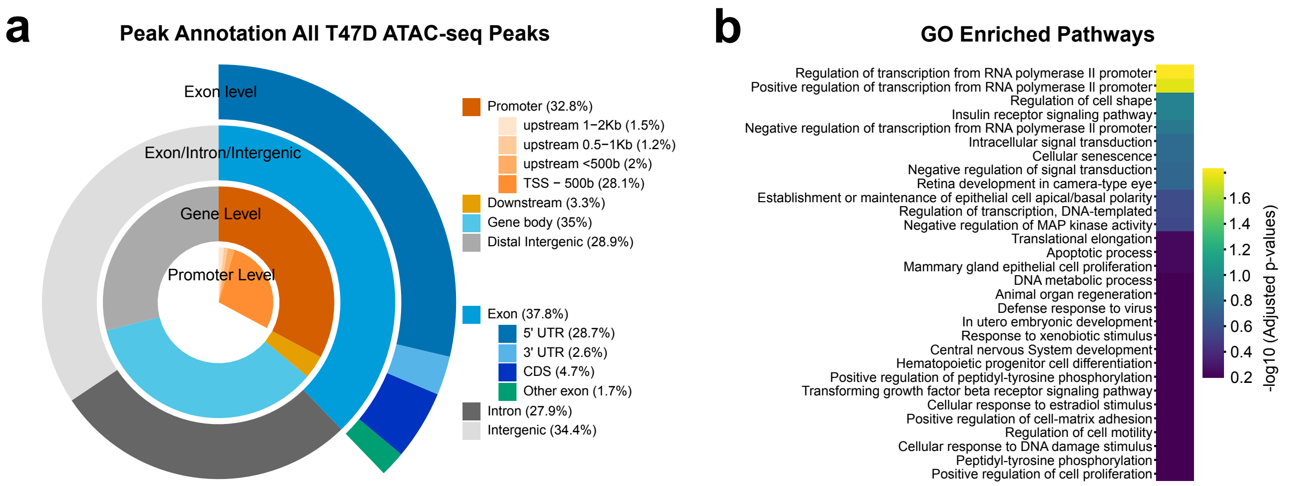

Supplementary Figure 2. Breast cancer cfDNA analysis using luminal breast cancer open chromatin.

a. Peak annotation of all T47D ATAC-seq peaks. Peaks are categorized into various groups, including promoter, exon, intron, and intergenic regions. **b.** Gene ontology (GO) analysis. Differentially enriched regions from breast cancer cfDNAs were assigned to nearest genes and GO biological process pathway analysis was conducted to identify associated biological pathways.

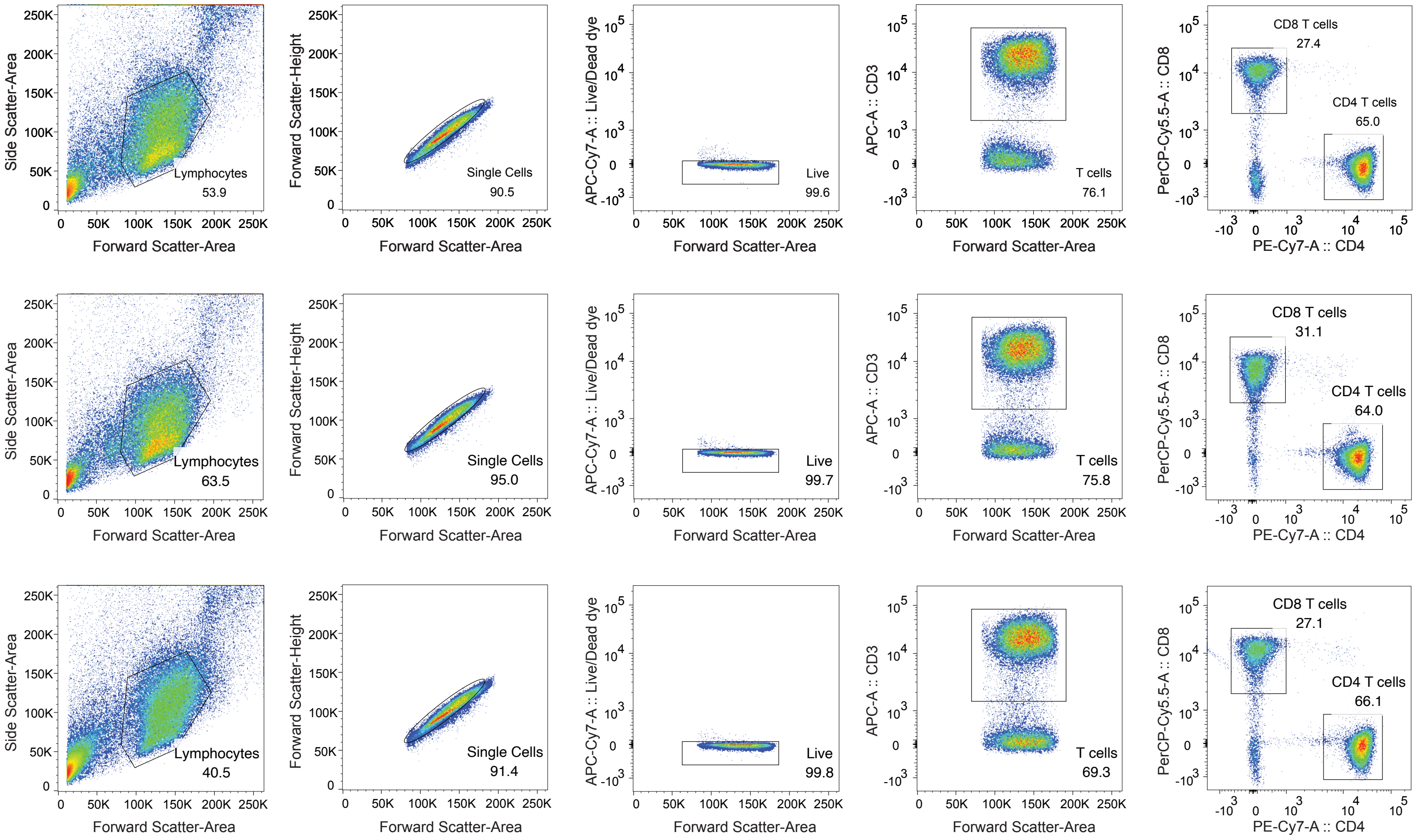

Supplementary Figure 3. Flow cytometry analysis of $CD4^+$ T cell frequencies in peripheral blood mononuclear cells (PBMCs) from breast cancer patients.

FACS gating strategy is shown for lymphocytes, singlets, and live PBMCs (left panels), followed by $CD3^+$ T cells and $CD4^+/CD8^+$ T cell subsets (right panels). Among the six breast cancer patient samples analyzed, representative results from three samples are displayed. Gates were applied sequentially to isolate $CD4^+$ and $CD8^+$ T cells, and their frequencies are indicated in the corresponding panels.

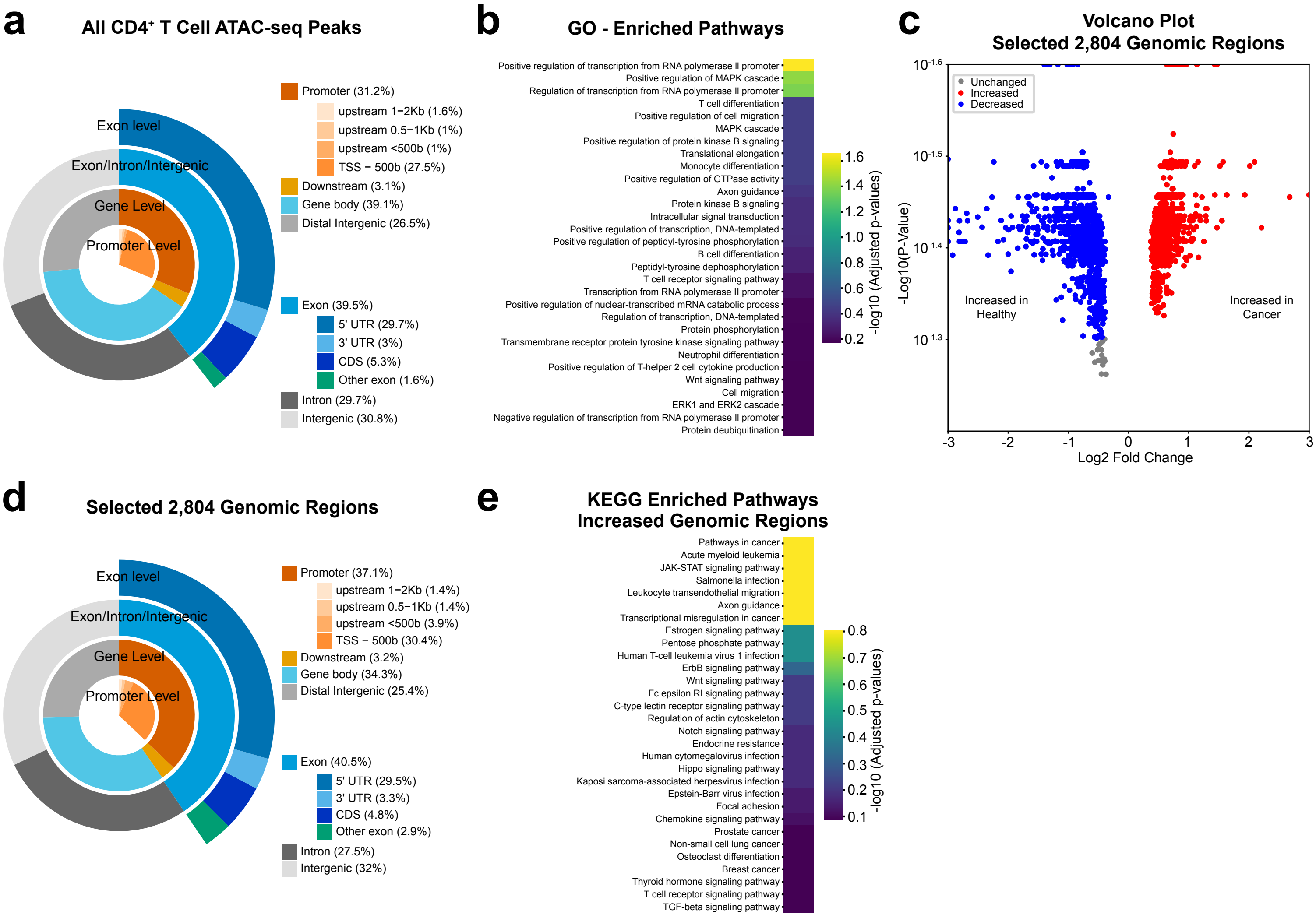

Supplementary Figure 4. cfNuc enrichment analysis using both luminal and immune cell ATAC-seq peaks.

a. Peak annotation of CD4⁺ T cell ATAC-seq peaks. Nested pie chart showing the distribution of ATAC-seq peaks across different genomic regions such as promoters and gene bodies. **b.** Top significant GO pathways associated with differential loci found at CD4⁺ T cell ATAC-seq peaks. **c.** Volcano plot showing cfNuc signal differences at the union (2,804) differential peaks. **d.** Peak annotation of 2,804 union genomic regions. **e.** KEGG pathways enrichment analysis of genes associated with decreased cfDNA signals in luminal breast cancer.

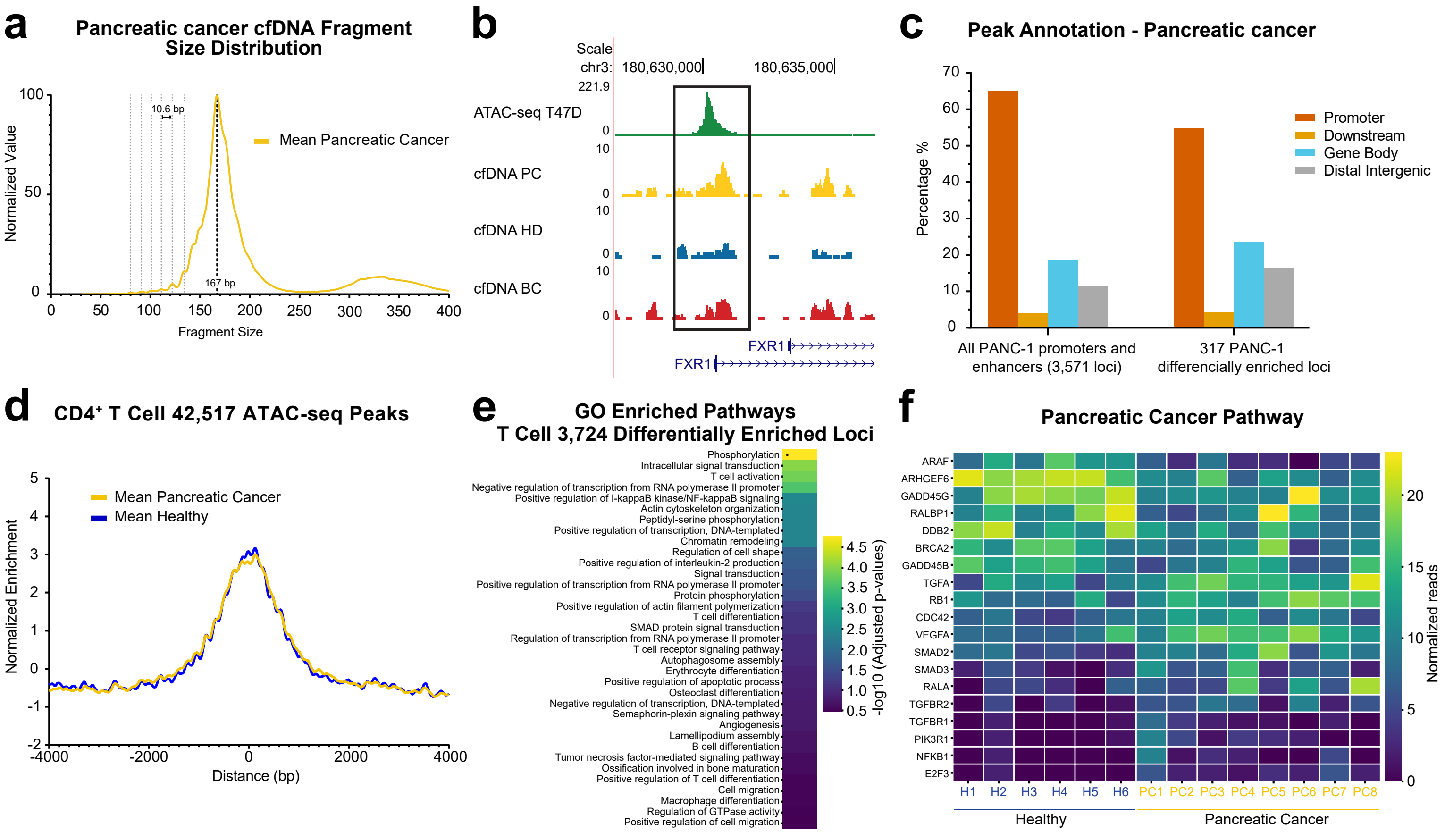

Supplementary Figure 5. Pancreatic cancer cfDNA analysis.

a. Fragment Size Distribution of cfDNAs isolated from pancreatic cancer patient plasma. **b.** Genome browser tracks of cfNuc signals from pancreatic cancer (PC), healthy donor (HD), and breast cancer (BC) samples. ATAC-seq T47D cells is shown as a reference for open chromatin regions in breast cancer cells. **c.** Peak annotation of all PANC-1 promoters and enhancers (left), and differential peaks found in cfDNAs derived from pancreatic cancer patients (right). **d.** Metaplot showing mean cfDNA enrichment in pancreatic cancer patients at CD4+ T cell ATAC-seq peaks. **e.** GO pathway analysis from 3,724 differential peaks found at CD4+ T cell ATAC-seq peaks. **f.** Heatmap showing expression levels of genes associated with pancreatic cancer pathway. Normalized read counts collected from each CD4+ T cell ATAC-seq peak are depicted as gene expression values.

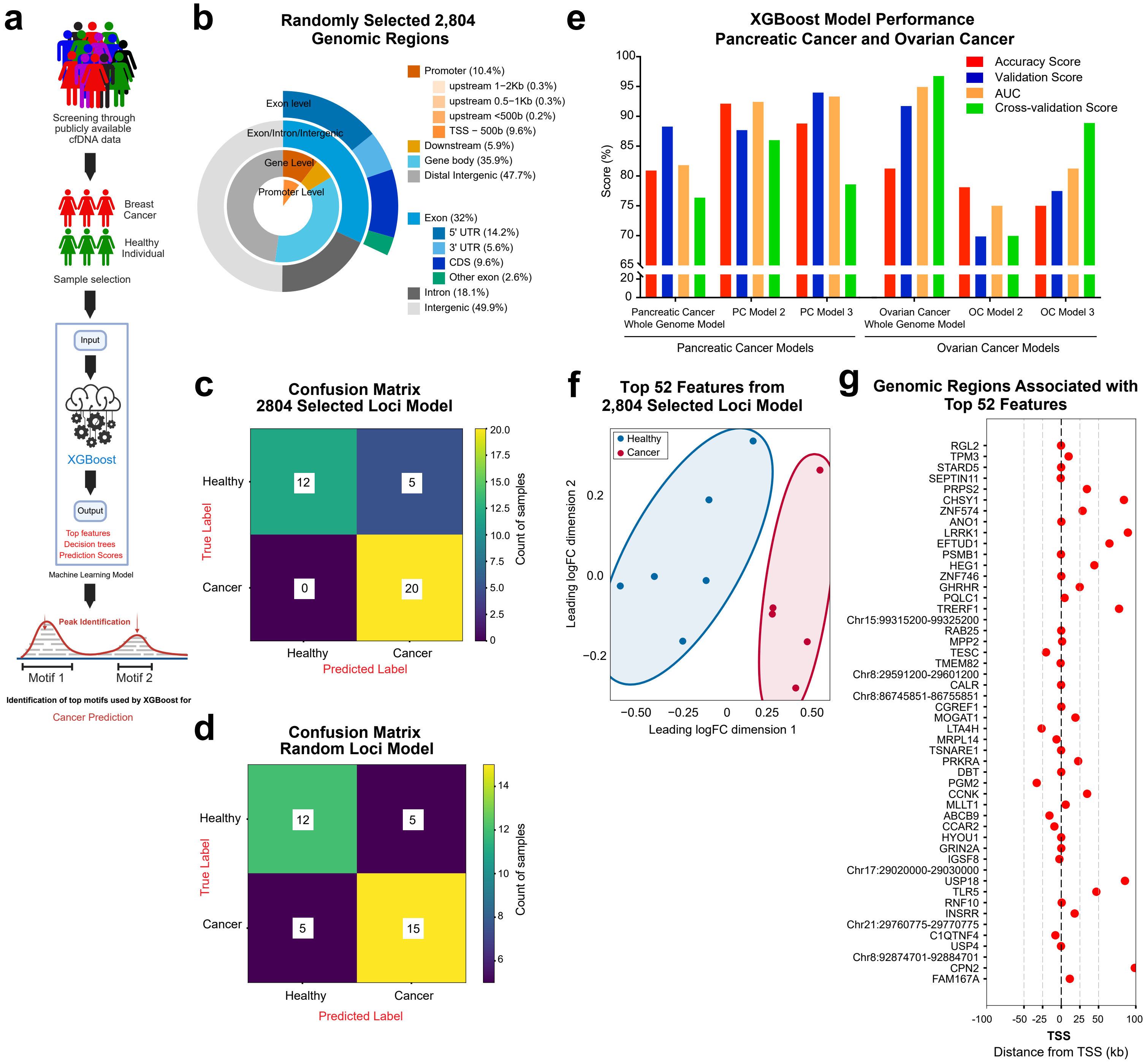

Supplementary Figure 6. Predictive analysis and feature interpretation using XGBoost machine learning.

a. Sample selection and data processing workflow for cfDNA analysis using XGBoost. **b.** Peak annotation of 2,804 randomly selected regions. Compared to 2,804 differential peaks found in breast cancer cfDNAs, promoter regions are slightly less frequent. **c.** Confusion matrix showing prediction results from an XGBoost-trained model. The model was established using publicly available cfDNA data, focused exclusively on 2,804 differential loci found in breast cancer cfNuc data. **d.** Confusion matrix from the XGBoost-trained model using randomly selected genomic loci. **e.** Comparison of XGBoost model performance for pancreatic and ovarian cancer cfDNA prediction. For each cancer type, three models were evaluated: Model 1 used whole-genome coverage data segmented into 10 kb bins; Model 2 used 10 kb-expanded regions derived from cancer-matched open chromatin regions (pancreatic or ovarian); and Model 3 used 10 kb-expanded regions combining cancer-matched open chromatin and CD4^{+} T cell ATAC-seq peaks. Bar graphs display prediction accuracy, validation score, AUC, and cross validation score for each model. Detailed results are summarized in Supplementary Table 3. **f.** MDS plot demonstrating clear separation of cfDNA profiles between healthy and breast cancer samples. Our cfDNA data were used for validation. Read counts were collected in the top 52 features identified by the XGBoost model. **g.** Distribution of the top 52 genomic features relative to TSS.

Supplementary Table 1. Clinical and demographic information of patients and healthy donors.

Figure Label	Material Type	Volume (ul)	Cancer/Control	Age	Sex	Stage	Collection Timing
BC1	PLASMA	600	Breast Cancer	78	F	Stage 1A, ER+ HER2-	Sample collected after surgery and radiation and Armidex
BC2	PLASMA	600	Breast Cancer	73	F	Stage I, grade 2, ER+/PR+/HER2-	Sample collected after Surgery and Adriamycin and Cytoxan, followed by paclitaxel and adjuvant radiation therapy
BC3	PLASMA	600	Breast Cancer	60	F	Stage 1A, ER+ HER2-	Sample collected after surgery/treatment.
BC4	PLASMA	600	Breast Cancer	65	F	Stage 0	Sample collected after radiation, tamoxifen
BC5	PLASMA	600	Breast Cancer	71	F	Stage 1A	Sample collected after surgery, radiation and aromatase inhibitor
H1	PLASMA	600	Healthy Donor	70	F	N/A	N/A
H2	PLASMA	600	Healthy Donor	61	F	N/A	N/A
H3	PLASMA	600	Healthy Donor	55	F	N/A	N/A
H4	PLASMA	600	Healthy Donor	34	F	N/A	N/A
H5	PLASMA	600	Healthy Donor	72	F	N/A	N/A
H6	PLASMA	600	Healthy Donor	48	F	N/A	N/A
PC1	PLASMA	600	Pancreatic Cancer	51	F	Stage 2A	Post treatment (Folfirinox, 5-FU Pump). No surgery.
PC2	PLASMA	600	Pancreatic Cancer	73	F	"Low grade" at time of collection. Developed stage 1B 2 years post specimen collection.	Pre surgery/treatment.
PC3	PLASMA	600	Pancreatic Cancer	70	F	Stage 1A	Post surgery. No treatment.
PC4	PLASMA	600	Pancreatic Cancer	69	M	Stage IV	Pre surgery. Post treatment (Abraxane and Gemzar).
PC5	PLASMA	600	Pancreatic Cancer	62	M	Stage IV	Post surgery. Post treatment (radiation, Xeloda, Abraxane/Gemcitabine, and Folfirinox)
PC6	PLASMA	600	Pancreatic Cancer	68	M	Stage IV	Post treatment (Abraxane/gemcitabine, Xeloda, Folfirinox, Oxaliplatin, 5FU/Onivyde). No surgery
PC7	PLASMA	600	Pancreatic Cancer	82	M	Stage 1B	Post treatment (Gemcitabine/Abraxane, Folfox). Pre surgery.
PC8	PLASMA	600	Pancreatic Cancer	67	M	Stage III	Post treatment (radiation, Xeloda, Folfirinox, Gemcitabine/Abraxane). Pre surgery.

Supplementary Table 2. XGBoost model parameters and performance summary.

**Model Name**	**2804 Significant Peak Model**	**Random 2804 Peak Model**	**Model 1**	**Model 2**	**Model 3**	**Model 4**	**Pancreatic Cancer Whole genome Model**	**PC Model 2**	**PC Model 3**	**Ovarian Cancer Whole Genome Model**	**OC Model 2**	**OC Model 3**
**Model Info**												
Peak Type	EdgeR identified 2804 Significant Peaks	Random 2804 Peaks	Whole Genome	T47D ATAC-Seq Peaks	CD4+ ATAC-Seq Peaks	Merged T47D and CD4+ Peaks	Whole Genome	PANC-1 promoters and Enhancers	PANC-1 Enhancers and Promoters Merged with CD4+ ATAC-Seq Peaks	Whole Genome	PEO1 ATAC-Seq peaks	PEO1 ATAC-Seq Peaks Merged with CD4+ ATAC-Seq peaks
Peak Bin Size	10kb	10kb	10kb	10kb	10kb	10kb	10kb	10kb	10kb	10kb	10kb	10kb
Model Type	Breast Cancer Vs Healthy Donor	Breast Cancer Vs Healthy Donor	Breast Cancer Vs Healthy Donor	Breast Cancer Vs Healthy Donor	Breast Cancer Vs Healthy Donor	Breast Cancer Vs Healthy Donor	Pancreatic Cancer Vs Healthy Donor	Pancreatic Cancer Vs Healthy Donor	Pancreatic Cancer Vs Healthy Donor	Ovarian Cancer Vs Healthy Donor	Ovarian Cancer Vs Healthy Donor	Ovarian Cancer Vs Healthy Donor
Number of cancer samples	64 (Female)	64 (Female)	64 (Female)	64 (Female)	64 (Female)	64 (Female)	34 (Male -18, Female - 16)	34 (Male -18, Female - 16)	34 (Male -18, Female - 16)	27 Females	27 Females	27 Females
Number of healthy samples	57 (Female)	57 (Female)	57 (Female)	57 (Female)	57 (Female)	57 (Female)	50 (Male - 25, Female - 25)	50 (Male - 25, Female - 25)	50 (Male - 25, Female - 25)	50 Females	50 Females	50 Females
Number of Features Used	2804	2804	Whole Genome	51463	42517	70499	Whole Genome	3571	28545	Whole Genome	55823	64757
**Hyperparameters**												
Best Learning Rate	0.44	0.4	0.1	0.39	0.37	0.64	0.55	0.63	0.41	0.35	0.35	0.63
Best Lambda (reg_lambda)	1	1	1	0	0	1	5	2	2	2	1	2
Early Stopping Rounds	15	15	15	15	15	15	15	15	15	15	15	15
Max Depth	6	6	4	6	6	6	6	6	6	6	6	6
Scale-pos-weight	1	1	1	0.89	0.89	0.89	1	1	1	1	1	1
**Model Performance**												
Balanced Accuracy	85.29%	72.79%	86.62%	82.79%	89.12%	92.06%	80.91%	92.12%	88.79%	81.25%	78.12%	75.00%
Validation AUC-PR	92.65%	82.06%	94.85%	94.46%	89.67%	97.14%	88.27%	87.68%	93.98%	91.72%	69.87%	77.48%
ROC AUC Score	92.00%	79.00%	92.00%	93.00%	90.00%	96.00%	81.82%	92.42%	93.33%	94.92%	75.00%	81.25%
Best CV Fold	3	3	3	3	3	3	6	4	8	8	8	8
Best CV AUC-PR	84.34%	84.33%	92.25%	88.00%	88.08%	89.04%	76.37%	86.01%	78.59%	96.76%	69.96%	88.89%
