# Supplementary material for: Open chromatin-guided interpretable machine learning reveals cancer-specific chromatin features in cell-free DNA
Source: Commun Biol. 2025 Nov 12;8:1554. doi: 10.1038/s42003-025-08920-0 (PMC12612109; doi:10.1038/s42003-025-08920-0)
Supplement: Supplementary file 2 — Description of Additional Supplementary Materials [file 42003_2025_8920_MOESM2_ESM.pdf]

Description of Additional Supplementary Files

File name: Supplementary Data 1

Description: List of 2,804 differentially enriched genomic regions.
